# Production and verification of a 2^nd^ generation clonal group of Japanese flounder, *Paralichthys olivaceus*

**DOI:** 10.1038/srep35776

**Published:** 2016-10-21

**Authors:** Jilun Hou, Guixing Wang, Xiaoyan Zhang, Yufen Wang, Zhaohui Sun, Fei Si, Xiufeng Jiang, Haijin Liu

**Affiliations:** 1 Beidaihe Central Experiment Station, Chinese Academy of Fishery Sciences, Qinhuangdao 066100, China

## Abstract

Clonal fishes are useful tools in biology and aquaculture studies due to their isogenicity. In Japanese flounder (*Paralichthys olivaceus*), a group of homozygous clones was created by inducing meiogynogenesis in eggs from a mitogynogenetic homozygous diploid. As the clones reached sexual maturity, meiogynogenesis was again induced in order to produce a 2^nd^ generation clonal group of Japanese flounder. After 3 months, there were 611 healthy, surviving individuals. Twenty-four microsatellite markers, that covered all the linkage groups of Japanese flounder, were used to identify the homozygosity of the 2^nd^ generation clones; no heterozygous locus was detected. This indicates that the production of a 2^nd^ generation clonal group of Japanese flounder was successful. Restriction-site DNA associated sequencing at the genomic level also confirmed the homozygosity and clonality of the 2^nd^ generation clonal group. Furthermore, these 2^nd^ generation clones had a small coefficient of variation for body shape indices at 210 days of age and showed a high degree of similarity in body characteristics among individuals. The successful production of 2^nd^ generation clones has laid the foundation for the large-scale production of clonal Japanese flounder.

Laboratory animals are commonly used for research purposes in several fields, including medicine, biology, and environmental toxicology *etc*. These animals possess desirable characteristics such as a clear genetic background and genetic uniformity among individuals from the same family. The traditional approach to preparing laboratory animals is continuous full-sib mating for at least 20 generations^1^. At that time, the theoretical coefficient of inbreeding is *F* = 0.986^2^. Although the coefficient of inbreeding is close to 1, 2% of genetic variance remains within the family. By using the full-sib mating approach, hundreds of inbreeding lines of rodents have been established and used commercially^3^. Additionally, inbreeding lines of fish are established by using this method in a variety of fish species^4^.

For most fish species, the matured eggs are spawned out of the body to be fertilized by sperm in the water when they are in the metaphase of second meiosis^5^. These reproductive traits make it possible to artificially induce polyploidy, androgenesis, and gynogenesis. Gynogenesis is a special mode of reproduction in which the homologous or heterologous spermatozoa enters the egg only to activate embryonic development. The sperm cannot develop into a male pronucleus and fuse with the female pronucleus to form a zygote. Offspring that hatch as a result of gynogenesis inherit genetic information only from the female parent^6^. When artificially inducing gynogenesis in fish, the egg is first activated by an irradiated homologous or heterologous sperm, and then the diploidy of the egg is restored via physical or chemical treatment. If the release of the second polar body is inhibited, meiogynogenesis is induced. This will result in the offspring being heterozygous due to the exchange of sister chromatids during meiosis. If cleavage is inhibited, mitogynogenesis is induced. Offspring that hatch as a result of mitogynogenesis is double haploid (DH) and have 100% homozygosity. The use of eggs or sperm that are spawned by the DHs to induce meiogynogenesis (eggs) or androgenesis (sperm) could establish a clonal line that has a coefficient of inbreeding of *F* = 1.00. Compared with full-sib mating, use of the chromosome manipulation method can establish a clonal line that has full homozygosity in short a period of time (2 generations). By using this method, clonal lines were successfully established in zebrafish (*Danio rerio*)^7^,^8^, medaka (*Oryzias latipes*)^9^, common carp (*Cyprinus carpio*)^10^,^11^, Nile tilapia (*Oreochromis niloticus*)^12^,^13^, amago salmon (*Oncorhynchus rhodorus*)^14^,^15^, ayu (*Plecoglossus altivelis*)^16^, rainbow trout (*Oncorhynchus mykiss*)^17^,^18^,^19^, Japanese flounder (*Paralichthys olivaceus*)^20^ and red seabream (*Pagrus major*)^21^.

To our knowledge, there is only one other report of a 2^nd^ generation of clonal fish by Müller-Belecke and Hörstgen-Schwark, who studied the survival rate, reproductive traits, and mean body weight of a 2^nd^ generation clones of tilapia^22^. A clonal line of Japanese flounder was established for the first time in China^23^. Because all individuals of the clonal line are females, it is impossible to produce 2^nd^ generation clones by male and female mating. Here, we describe the creation of a 2^nd^ generation clonal group of Japanese flounder by induced meiogynogenesis using eggs spawned by 1^st^ generation clones (Fig. 1).

## Results

### Induction rate of 2^nd^ generation clones

In total, five out of 27 1^st^ generation clones were used for the meiogynogenetic induction to create the 2^nd^ generation clones. The cleavage rate, hatching rate, and abnormal rate was calculated for three of them. The control group consisted of eggs from the 1^st^ generation clones that were fertilized by sperm from wild-type males (three males) or double haploid males (three males, and the progeny are heterozygous clones). Due to the poor quality of eggs spawned by the clones, a wide variety of low values were obtained for cleavage, hatching, and abnormal rates. The abnormal rate was higher in the meiogynogenesis group than in the other two groups (Table 1). This may have been caused by the side effects of the cold-shock treatment. By the age of 3 months, we had 611 healthy, surviving individuals.

### Homozygosity analysis by microsatellite genotyping

**Figure 2:**
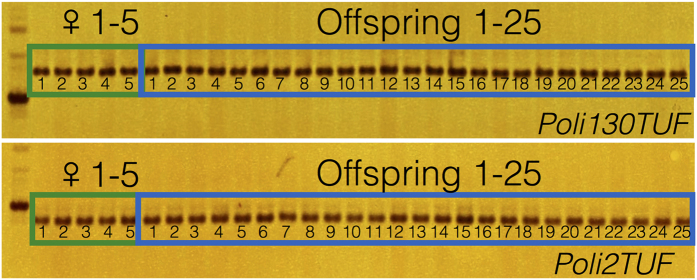
Electrophorogram of PCR products for *Poli130TUF* and *Poli2TUF* microsatellite loci in 1^st^ and 2^nd^ generation clones of Japanese flounder (*Paralichthys olivaceus*). ♀1–5: 1^st^ generation clone individual 1 to 5; Offspring 1–25: 2^nd^ generation clone individual 1–25. Note each individual had the only one allele and same allele size at each locus, indicating the clonality and homozygosity of each samples.

The homozygosity of the 2^nd^ generation clonal group and the genetic similarities between the 1^st^ and 2^nd^ generation clones were analyzed by using 24 microsatellite markers that cover the 24 linkage groups of Japanese flounder. In five of the 1^st^ generation clones, each individual had the same allele size at each locus, and no heterozygous loci were detected. These results represent the clonality and homozygosity of the 1^st^ generation clones. The 25 2^nd^ generation clones had the same band pattern as the 1^st^ generation clones, and only homozygous loci were detected (Table 2, Fig. 2). The microsatellite genotyping results indicated that the production of the 2^nd^ generation clones was successful.

### Homozygosity analysis by Restriction-site associated DNA sequencing

A total of 10.571 Gb of raw data was obtained from the five individuals that were sequenced (submitted to SRA database, accession No. SRP087604). The raw data from each individual ranged from 1283.039 Mb to 4556.052 Mb. After filtering through the data, 10.403 Gb of clean data was selected for further analyses. All samples were of high quality (Q20 ≥ 93.13%, Q30 ≥ 85.0%), and the GC contents ranged from 39.75% to 40.26% (Table S1). The 1^st^ generation clone that was used to assemble the reference sequence obtained 10851243 digestion reads (RAD tags). The other four samples had RAD tags ranging from 3340795 to 4425361 (Table S2).

The RAD reference sequence that was assembled using 1^st^ generation clone sequences contained 212820 contigs with 78774559 bp. The average contig length was 370 bp (Table S3, Figure S1). This covered approximately of the sequenced genome of Japanese flounder. To evaluate accuracy of the assembled reference sequence, the high-quality pair-end reads (125-bp) were realigned onto the assembled scaffolds. An average depth of 24.92 was obtained, and approximately 89.54% of the reference assembly was covered by 4 or more reads (Table S3).

For all five samples, the alignment rates to reference sequence ranged from 66.28% to 72.36%, and the average depth (excluding the N region) was between 8.44X ~24.43X (Table S4). The total SNP loci ranged from 304129 to 309307, and the control had 190010 heterozygous SNPs. However, the 1^st^ generation clone and three 2^nd^ generation clones, had heterozygous SNP numbers that were only 5218 to 6715. These were significantly smaller than the control (Tables 3 and S5). The heterozygosity rate was calculated as the ratio of heterozygous SNPs to total SNPs. The results indicated that the 1^st^ and 2^nd^ generation clones had low heterozygosity rates (1.72%~2.17%), and the control had the heterozygosity rate of 61.83% (Table 3). Due to the homozygous pairing of alleles during meiogynogenesis, the heterozygosity rates of three of the 2^nd^ generation clones were lower than those of the 1^st^ generation clones. The results revealed that the genetic similarity index between clones was above 0.9643 (Table 4). The low heterozygosity rate and high genetic similarity obtained from RAD sequencing indicate the successful production of 2^nd^ generation clones at the genomic level.

### Morphological characteristics

The ratios of total length/body length, total length/head length, total length/caudal peduncle length, total length/withers height, caudal peduncle length/caudal peduncle height, and withers height/caudal peduncle height of the 2^nd^ generation clonal group were significantly different from those of the control group (*P* < 0.01). Within the 2^nd^ generation clonal group, only the CV of the total length/caudal peduncle length ratio was higher than 3% (3.62%), the CVs of all other ratios were lower than 3%. However, within the control group, only the CV of the total length/body length ratio was lower than 3% (2.82%), the other ratios’ CVs were all higher than 3% (Table 5). These results revealed that the control group had higher CVs of morphological characteristics, and presented a large number of morphological differences between individuals within the family. In contrast, the 2^nd^ generation clonal group had low CVs, and individuals within the family had highly similar morphological characteristics.

## Discussion

In this study, using eggs from homozygous clones, the 2^nd^ generation clonal group was induced by artificial meiogynogenesis. This is the first time that 2^nd^ generation clones of Japanese flounder were induced by three cycles of gynogenesis (one cycle of mitogynogenesis and two cycles of meiogynogenesis).

The homozygosity and clonality of 2^nd^ generation clones were verified by microsatellite markers and RAD-Seq. Interestingly, 1.7–2% heterozygosity rate was detected in 1^st^ and 2^nd^ generation clones from the RAD-Seq results. Theoretically, the clone induced from doubled haploid by meiogynogenesis must be totally homozygous, and no heterozygous locus Should be detected. Sequencing error might be one possible explanation for the heterozygous SNPs. Random and systematic errors are inevitably for the common high-throughput sequencing platforms^24^. Although there are a lot of programs that based on different data structures and algorithms available for error removal in sequencing data, it is impossible to remove all the errors due to each program’s limitation^25^. The other explanation for the heterozygous SNPs might be the meigynogenesis induction process. We used 0 °C cold-shock treatment to inhibit the releasing of second polar body. Such cold-shock treatment might cause the mismatch of bases (Non-complementary bases in a duplex DNA), and thus result in the heterozygous SNPs of clones. However, more studies are needed to clarify the mechanism of heterozygous SNPs appeared in homozygous clones.

Compared to the wild-type control group, the 2^nd^ generation clones had lower CV in ratios of morphological traits, and individuals possessed highly similar morphological characteristics. These ratios and CVs were not different from those of their clonal 120-day-old female parents (1^st^ generation clones) (unpublished data). The data concerning the morphological traits of the 1^st^ and 2^nd^ generation clones indicate that they are different from other Japanese flounder groups. Other groups have a high degree of consistency among ratios of morphological traits during the process of growth and intergeneration. The results of this study are consistent with those of similar studies. In mice, the homozygous strains and F_1_ hybrids displayed reduced CV for mandible length as compared to outbred or F_2_ progeny^26^. In the amago salmon^14^, ayu^16^, and Nile tilapia^22^, the same trend was found in the phenotypic variation between the homozygous clones and the crosses of outbred fish. However, In the present study, both 2^nd^ generation clone group and wild-type control group had increased CVs in body weight and total length. And for each of these two character, CV of 2^nd^ generation clone group was higher than the wild-type control group. In common carp, the homozygous clones had increased variations in body weight and length when compared to the crosses of homozygous clones and outbred males. The variation, however, was not significantly different from the outbred crosses when the homozygous clones were produced by crossing females with sex-reversed clonal males. The authors believe that the variation in the homozygous clones was partially caused by treatment effects^27^. However, more studies are needed to discriminate the factors that influence the phenotype variation in homozygous clones.

Clonal lines originating from androgenetic or mitogynogenetic DHs represent unique biological tools which can be used to identify important aquaculture industry parameters, such as feed uptake, growth rate, deformities, and disease resistance^3^. Several clonal lines of rainbow trout were established using gynogenesis or androgenesis, and the development rate of differences among traits, such as cytotoxic cell activity, were genetically dissected^28^,^29^. Quillet *et al*. found variations in susceptibility to rhabdoviruses within rainbow trout clonal lines, thereby these animals could be used to further investigate the genetic mechanism of resistance^19^. Crosses between different clonal lines can be used to produce heterozygous clones. Heterozygous clones are free of lethal recessive genes and often show heterosis, in terms of viability and growth related traits, relative to homozygotes^6^. In Japanese flounder, we produced several heterozygous clonal lines, and one of them displays high heterosis in growth rate, which is 77.66% faster than the controls (unpublished data). The induction of mitogynogenesis or androgenesis in the heterozygous clones can produce recombined DHs, which are valuable tools for gene mapping and for QTL detection^30^.

The homozygous clonal lines could also be used for the study of human diseases. Mizgirev and Revskoy produced homozygous clonal lines of zebrafish, and using these clonal lines, established the model of liver tumor cell transplantation^31^. This provided reference material for the continued study of tumor growth, angiogenesis, metastasis, and the therapeutic effect of anticancer drugs^31^. In a comparative study of hematological indices between homozygous clones and common Japanese flounder, aspartate aminotransferase (AST) was significantly higher in clonal Japanese flounder than in common Japanese flounder (*P* < 0.01). This revealed that these homozygous clones could be used as a liver disease model^32^.

As environmental issues have become increasingly prominent, the study of the impact of environmental pollution has become more important. Fish are widely used in the study of the water pollution^33^. The degree of standardization among the animals used in the study has a direct impact on the accuracy and repeatability of the results. In studies on marine pollution, the majority of experimental animals used were non-standard fish. The genetic background of these fish was not clear, and the genetic similarities were low, which reduced the repeatability of the results. The use of standardized experimental animals in future research, can effectively overcome these problems, and improve the accuracy of the study. The 1^st^ generation homozygous clonal line that were utilized as female parents in this experiment were applied in the study of acute toxicity of Hg^2+^. The tolerance and consistency of death between clonal and common Japanese flounder were compared. The results indicated that the clones were more sensitive to Hg^2+^, and had a higher consistency of death^34^. Currently, there is no standard marine experimental animal line available for use. However, the homozygous clonal Japanese flounder we produced could meet the criteria of an experimental animal, and could be used in studies of marine environmental pollution.

In conclusion, by the artificial induction of meiogynogenesis using eggs of homozygous clonal Japanese flounder, 2^nd^ generation homozygous clones were successfully created, and this has laid the foundation for the large-scale application of clonal Japanese flounder.

## Materials and Methods

### Ethics

This study was performed in accordance with the Guide for Care and Use of Laboratory Animals provided by the Chinese Association for Laboratory Animal Sciences (No. 2011–2). All the experiment protocols were approved by the animal care and use committee of Beidaihe Central Experiment Station.

### Fish and gamete collection

All the fishes we used in this study were 4 year old, and reared at the Beidaihe Central Experiment Station, Chinese Academy of Fishery Sciences, Qinhuangdao, Hebei Province. The sperm of red sea bream was collected using a 5-mL plastic syringe by gently pressing on the abdomen, thus avoiding water and urine, and stored in the dark at 4 °C until used. The sperm of Japanese flounder was collected via the same procedure. The eggs were collected by gently stripping the abdomen of female Japanese flounder into a 1000-mL glass beaker, and stored in the dark at room temperature.

### Induction of meiogynogenesis

The induction of meiogynogenesis was executed according to the procedure of Yamamoto (1999)^19^. The sperm of red sea bream was UV-irradiated with a dosage of 73 mJ/cm^2^, and then added to the eggs and mixed well. The eggs were activated by adding marine water (17 °C). Three minutes after activation, the eggs were subjected to a 0 °C cold-shock treatment for 45 min. After the cold-shock treatment, the eggs were transferred to marine water (17 °C) for hatching. Totally, we induced five batches of meiogynogenesis using eggs from five 1^st^ generation clones.

### Preparation of control groups

Control groups were produced simultaneously with the meiogynogenetic groups. To evaluate cleavage, hatching, and abnormal rates, the eggs of clones were artificially fertilized with sperm of wild-type and DH males, and then reared at 17 °C. To measure morphological characteristics, eggs from one wild-type female were fertilized by one wild-type male.

### Cleavage, hatching and abnormal rates

The cleavage rate was calculated by finding the frequency of gastrula stage eggs as compared to the total number of eggs used. The occurrence of hatched larvae compared to the total number of eggs used provided the hatching rate. In addition, the frequency of abnormal larvae compared to the hatched larvae revealed the abnormal rate. For each group, more than 1,000 eggs were used to determine these calculations.

### Microsatellite genotyping

Fin clips from five female clones and 25 2^nd^ generation juvenile clones (from mixed population of 2^nd^ generation clones) were sampled, placed in 100% ethanol, and stored at −20 °C. Genomic DNA from each sample was isolated using phenol-chloroform extraction^35^.

In total, 24 microsatellite loci, that covered all 24 linkage groups of Japanese flounder, were randomly selected for genotyping^36^. PCR was performed in a 15-μl reaction cocktail containing 40–50 ng template DNA, 1X PCR buffer (50 mM of KCl, 10 mM of Tris–HCl, 1.5 mM of MgCl_2_, pH 8.3), 200 μM of each dNTP, 1 U *Taq* polymerase (Takara), and 2 pmol of each primer under the following conditions: one cycle of initial denaturation for three min at 94 °C, 25 cycles of denaturation for 30 sec at 93 °C, annealing for 30 sec at 62 °C, extension for 30 sec at 72 °C, and one cycle of final extension for 10 min at 72 °C. The PCR products underwent electrophoresis and were analyzed further with the method of Liao *et al*.^37^.

### RAD sequencing

Fin clips of one female clone, three 2^nd^ generation clones and one control were sampled, placed in 100% ethanol, and stored at −20 °C. Genomic DNA from each sample was isolated using phenol-chloroform extraction^35^. The genomic DNA from each sample was digested by *EcoRI* and an adapter (P1) was ligated to the fragment’s compatible ends. The adapter-ligated fragments were subsequently pooled, randomly sheared, and size-selected. The fragments were then ligated to a second adapter (P2), a Y adapter that has divergent ends^38^. Finally, the fragments from the 18 cycles of PCR amplification of 200 bp to 400 bp were collected for the purpose of constructing a library. A Qubit2.0 kit was used to analyze the quality of the library. After the library was diluted to 1 ng/ul, Agilent 2100 was used to check the insert size of the library. Q-PCR was performed to determine the effective concentration of the library (the effective concentration of library >2 nM) when the insert size was appropriate. All of these steps were taken to ensure the quality of the library. The constructed library was sequenced using the Illumina HiSeq2500 platform at Novogene in Beijing, and 125-bp paired-end reads were generated.

Due to the unavailability of the presently existing genomic information of Japanese flounder, we specified 1^st^ generation clone to be sequenced with the greatest amount of data (approximately 5× whole-genome coverage for this reference individual; 1× whole-genome coverage for others) for sequence used as a reference for downstream analysis. The clean reads from the sequencing were obtained by removing reads containing adapter, poly-N, and low quality reads (bases that more than 50% of the single end sequencing read length are under score 5) from the raw data using FASTX-Toolkit (http://hannonlab.cshl.edu/fastx_toolkit/). The reference sequence was assembled by VelvetOptimiser assembler (settings: -s 23 -e 31 -x 4)^39^.

Sequence readings from all five samples were aligned with the reference sequence using BWA software (v0.7.8, settings: aln -o 1 -m 100000 -t 4 -l 32 -i 15 -q 10)^40^. Repeats from the alignment results were removed using SAMtools software (v0.1.19, settings: rmdup)^41^. The SNP detection for each sample was performed using the mpileup function in SAMtools (settings: mpileup -m 2 -F 0.002 -d 1000). The genetic similarity index was calculated using the formula presented by Nei & Li (1979)^42^.

### Measurement of morphological characteristics

The 2^nd^ generation clone group and control group were reared under the same culture conditions. Briefly, for each group, newly hatched larvae were reared in an aquarium (300 × 100 × 100 cm) with flow-through seawater. The fish were fed *Brachionus plicatilis* from day 0 to 25, *Artemia salina* from day 15 to 60 and standard commercial feed after day 60. Then, same number of individuals from each group were transferred to two 25-m^3^ tanks (one tank for one group) at day 61. During the experiment, the rearing density, feeding level, water temperature (fluctuated naturally from 17 to 22 °C) and flow velocity were maintained similar between 2^nd^ generation clone group and control group. At the age of 210 days, each 30 individuals from the 2^nd^ generation clone group and the control group, were randomly selected and measured for the following traits: total length, body length, head length, caudal peduncle length, withers height, and caudal peduncle height. After measurements had been collected, the following trait ratios were calculated: total length/body length, total length/head length, total length/caudal peduncle length, total length/withers height, caudal peduncle length/caudal peduncle height, and withers height/caudal peduncle height.

### Statistical analysis

The data is given in the format of Mean ± SD (standard deviation). The coefficient of variation (CV) was calculated as the ratio of SD to Mean. Paired t-tests were performed using R software^43^ to compare the morphological characteristics between the 2^nd^ generation clonal group and the wild-type control.

## Additional Information

**How to cite this article**: Hou, J. *et al*. Production and verification of a 2^nd^ generation clonal group of Japanese flounder, *Paralichthys olivaceus. Sci. Rep.* **6**, 35776; doi: 10.1038/srep35776 (2016).

## Supplementary Material

Supplementary Information

Supplementary Table S5

## Figures and Tables

**Figure 1:**
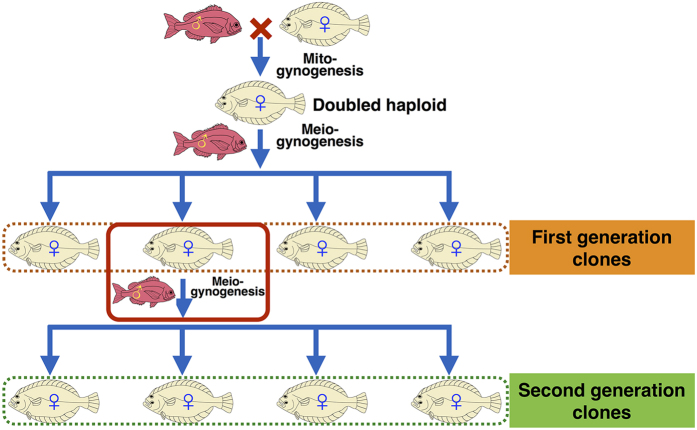
The schematic flow of the production procedure of 2^nd^ generation clones of Japanese flounder (*Paralichthys olivaceus*).

**Table 1:** Cleavage, hatching and abnormal rates of different treatment groups in Japanese founder (*Paralichthys olivaceus*).

Female	Male	Treatment	Total eggs	Cleavage (%) (Range)	Hatching (%) (Range)	Abnormal (%) (Range)
1^st^ clone	Wild-type	Normal fertilization	4754	33.67 (0.38~77)	16.97 (0.23~48.39)	5.54 (0~11.11)
1^st^ clone	Doubled Haploid	Normal fertilization	2488	43.93 (24.05~81.85)	30.54 (6.94~61.17)	8.37 (5.08~14.85)
1^st^ clone	UV-irradiated red sea bream sperm	3 min AA*, 0 °C, 45 min	5708	20.40 (5.75~45.11)	11.29 (0.51~28.78)	22.88 (6.35~55.56)

^*^AA, after activation.

**Table 2:** Microsatellite genotyping results of clonal female parents and 2^nd^ generation clones in Japanese founder (*Paralichthys olivaceus*).

Linkage group	Locus^a^	*♀*1–5	Offspring 1–25	Linkage group	Locus^a^	*♀*1–5	Offspring 1–25	Linkage group	Locus^a^	*♀*1–5	Offspring 1–25
1	*Poli130TUF*	*127/127*	*127/127*	9	*Poli182TUF*	*118/118*	*118/118*	17	*Poli1407TUF*	*166/166*	*166/166*
2	*Poli866TUF*	*258/258*	*258/258*	10	*Poli101TUF*	*147/147*	*147/147*	18	*Poli16–79TUF*	*130/130*	*130/130*
3	*Poli188TUF*	*136/136*	*136/136*	11	*Poli174TUF*	*137/137*	*137/137*	19	*Poli1490TUF*	*173/173*	*173/173*
4	*Poli148TUF*	*197/197*	*197/197*	12	*Poli212TUF*	*145/145*	*145/145*	20	*Poli139TUF*	*145/145*	*145/145*
5	*Poli9TUF*	*169/169*	*169/169*	13	*Poli966TUF*	*172/172*	*172/172*	21	*Poli1925TUF*	*221/221*	*221/221*
6	*Poli107TUF*	*151/151*	*151/151*	14	*Poli141TUF*	*176/176*	*176/176*	22	*Poli2TUF*	*124/124*	*124/124*
7	*Poli18–55TUF*	*120/120*	*120/120*	15	*Poli39MHFS*	*221/221*	*221/221*	23	*Poli193TUF*	*127/127*	*127/127*
8	*Poli1825TUF*	*145/145*	*145/145*	16	*Po25A*	*224/224*	*224/224*	24	*Poli607TUF*	*152/152*	*152/152*

^a^See Castaño-Sánchez *et al*.^36^.

**Table 3:** Summary of SNP number and heterozygous rate of each sample in Japanese founder (*Paralichthys olivaceus*).

Sample	Total SNP	Heterozygous SNP	Heterozygous rate (%)
1^st^ generation clone	309307	6715	2.17
2^nd^ generation clone-1	304129	5218	1.72
2^nd^ generation clone-2	305719	5349	1.74
2^nd^ generation clone-3	306359	5877	1.92
Control	307308	190010	61.83

**Table 4:** The genetic similarity index between samples in Japanese founder (*Paralichthys olivaceus*).

	1^st^ generation clone	2^nd^ generation clone-1	2^nd^ generation clone-2	2^nd^ generation clone-3	Control
1^st^ generation clone	*****	0.9644	0.9683	0.9720	0.0982
2^nd^ generation clone-1	0.9644	*****	0.9649	0.9643	0.0940
2^nd^ generation clone-2	0.9683	0.9649	*****	0.9677	0.0952
2^nd^ generation clone-3	0.9720	0.9643	0.9677	*****	0.0963
Control	0.0982	0.0940	0.0952	0.0963	*****

**Table 5:** The ratios of morphological traits of second generation clones and wild-type control.

Traits Family	Body weight (g)		Total length (cm)		Total length/body length		Total length/head length		Total length/caudal peduncle length		Total length/withers height		Caudal peduncle length/caudal peduncle height		Withers height/caudal peduncle height	
	Mean ± SD	CV%	Mean ± SD	CV%	Mean ± SD	CV%	Mean ± SD	CV%	Mean ± SD	CV%	Mean ± SD	CV%	Mean ± SD	CV%	Mean ± SD	CV%
2^nd^ generation clones	53.86 ± 14.90^b^	27.66	17.04 ± 1.68^b^	9.83	1.19 ± 0.03^a^	2.48	4.12 ± 0.06^a^	1.36	12.89 ± 0.47^a^	3.62	3.10 ± 0.07^a^	2.39	0.97 ± 0.02^a^	2.29	3.99 ± 0.07^a^	1.66
Wild-type control	145.48 ± 38.41^a^	26.40	25.03 ± 2.01^a^	8.05	1.15 ± 0.03^b^	2.82	4.30 ± 0.17^b^	3.88	11.50 ± 1.85^b^	16.11	2.96 ± 0.09^b^	3.01	1.09 ± 0.16^b^	14.64	4.14 ± 0.18^b^	4.24

Note: different superscript letters within columns indicate significant differences as determined by paired t-tests (*P* < 0.01).
