# Supplementary material for: Production and verification of a 2nd generation clonal group of Japanese flounder, Paralichthys olivaceus
Source: Sci Rep. 2016 Oct 21;6:35776. doi: 10.1038/srep35776 (PMC5073307; doi:10.1038/srep35776)
Supplement: Supplementary Information [file srep35776-s1.pdf]

Title: Production and verification of a 2nd generation clonal group of Japanese flounder, *Paralichthys olivaceus*

Jilun Hou[1, †], Guixing Wang[1, †], Xiaoyan Zhang[1], Yufen Wang[1], Zhaohui Sun[1], Fei Si[1], Xiufeng Jiang[1] & Haijin Liu[2]

[1]Beidaihe Central Experiment Station, Chinese Academy of Fishery Sciences, Qinhuangdao 066100, China

[2]Centre for Applied Aquatic Genomics, Chinese Academy of Fishery Sciences, Beijing, 100141, China

Correspondence should be addressed to J.H. (email: jilunhou@hotmail.com) and H.L. (email: liuhaijin2005@126.com)

Table S1 Summary of sequencing data.

Sample	Raw Base(bp)	Clean Base(bp)	Effective Rate(%)	Error Rate(%)	Q20(%)	Q30(%)	GC Content(%)
1st generation clone	4,556,052,000	4,547,654,400	99.82	0.03	95.86	91.25	40.12
2nd generation clone-1	1,283,038,500	1,176,728,700	91.71	0.07	93.21	85.00	40.26
2nd generation clone-2	1,326,043,800	1,283,231,400	96.77	0.06	93.13	85.00	40.03
2nd generation clone-3	1,690,509,600	1,685,015,100	99.67	0.03	96.30	92.05	39.75
Control	1,715,690,400	1,710,505,800	99.70	0.03	96.25	91.95	39.84

Table S2 Summary of obtained restriction-site associated DNA tags.

Sample	Clean reads	Removed duplication reads	Clean duplication rate(%)	Digestion reads	Digestion ratio(%)
1st generation clone	15,158,848	11,192,166	26.17	10,851,243	96.95
2nd generation clone-1	3,922,429	3,690,307	5.92	3,340,795	90.53
2nd generation clone-2	4,277,438	3,969,574	7.20	3,879,550	97.73
2nd generation clone-3	5,616,717	4,439,487	20.96	4,313,060	97.15
Control	5,701,686	4,555,744	20.10	4,425,361	97.14

Table S3 Summary of restriction-site associated DNA tags assembly of 1st generation clone.

Total contig base (bp)	78774559	GC(%)	40.70
Total contig number	212820	Average depth	24.92
Average contig length (bp)	370	Mapping rate (%)	86.78
N50 length (bp)	431	Coverage (%)(at least 4×)	89.54

Table S4 Summary of sequencing depth and coverage for the 5 samples that were aligned with reference sequence (1st generation clone).

Sample	Mapped reads	Total reads	Mapping rate(%)	Average depth(X)	Coverage at least 1X(%)	Coverage at least 4X(%)
1st generation clone	21937575	30317696	72.36	24.43	98.41	87.22
2nd generation clone-1	5199693	7844858	66.28	8.44	84.99	61.92
2nd generation clone-2	6129600	8554876	71.65	9.71	85.84	66.03
2nd generation clone-3	8029814	11233434	71.48	11.19	87.38	69.12
Control	7681860	11403372	67.36	10.96	84.06	65.74

Table S5: SNP genotypes results of each samples after restriction-site associated DNA sequencing.

Please see the separate Excel document.

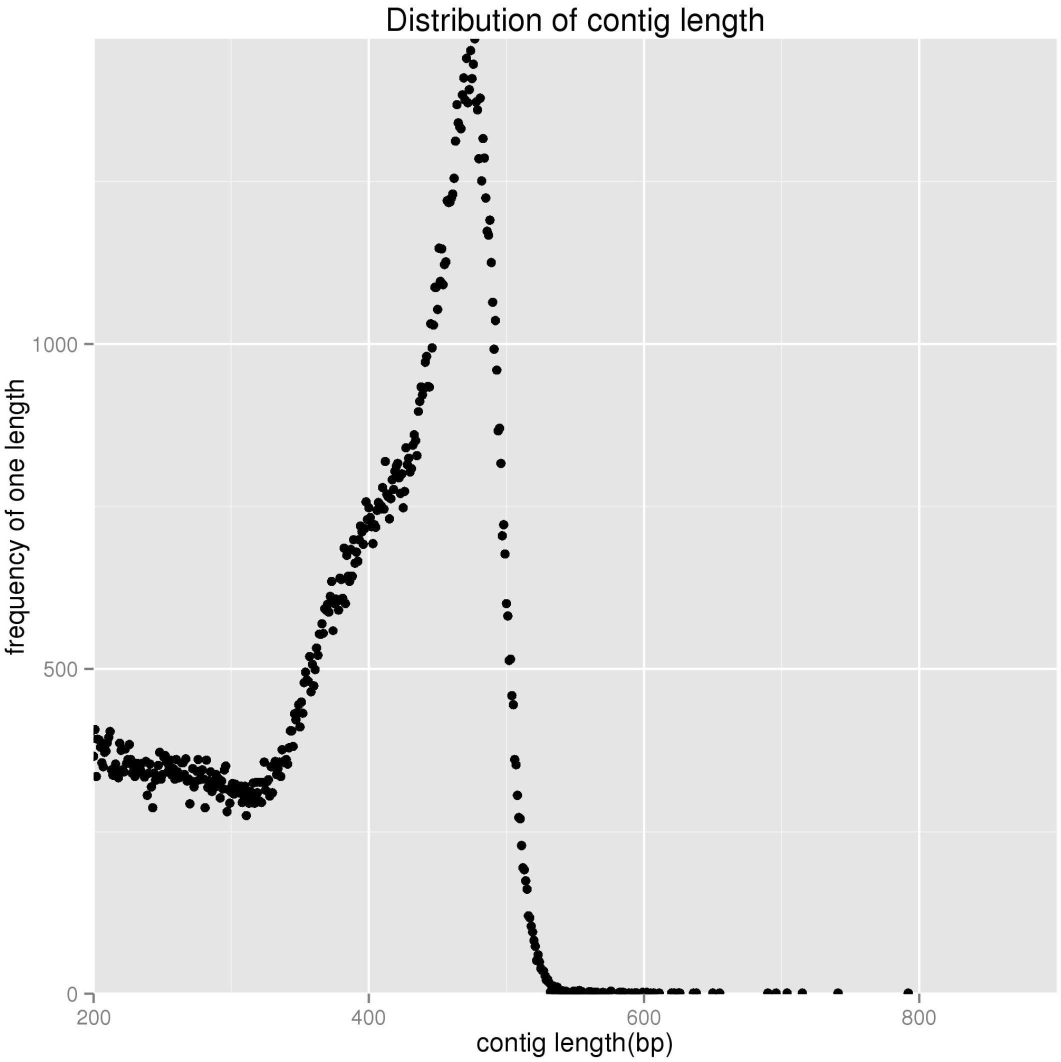

Figure S1: The distribution of assembled contig length of 1st generation clone's restriction site-associated DNA sequences that were used as the reference sequence.
